# Lexis in Chinese-English Translation of Drug Package Inserts: Corpus-based Error Analysis and Its Translation Strategies

**Published:** 2010-12

**Authors:** Lin Ying, Zhou Yumei

**Affiliations:** *Department of Foreign Languages, the Fourth Military Medical University, Xi’an, China*

**Keywords:** drug package insert, translation, error analysis, lexical error, strategy

## Abstract

Error analysis (EA) has been broadly applied to the researches of writing, speaking, second language acquisition (SLA) and translation. This study was carried out based on Carl James’ error taxonomy to investigate the distribution of lexical errors in Chinese-English (C-E) translation of drug package inserts (DPIs)(1), explore the underlying causes and propose some translation strategies for correction and reduction of lexical errors in DPIs. A translation corpus consisting of 25 DPIs translated from Chinese into English was established. Lexical errors in the corpus and the error causes were analyzed qualitatively and quantitatively. Some examples were used to analyze the lexical errors and their causes, and some strategies for translating vocabulary in DPIs were proposed according to Eugene Nida’s translation theory. This study will not only help translators and medical workers reduce errors in C-E translation of vocabulary in DPIs and other types of medical texts but also shed light on the learning and teaching of C-E translation of medical texts.

## INTRODUCTION

With the increasing exchanges of Chinese pharmaceutical industry with other countries, accurate and smooth Chinese-English translation of a drug package insert (DPI) is becoming more and more important. It can not only instruct the patients to use the drug rationally but also enhance the reliability of the overseas medical workers and patients on Chinese exported drugs.

During the past decades quite a few domestic and overseas scholars have conducted researches into translation of DPIs and have made some remarkable achievements (Ma Bangxin, 1998; Montalt & Davies, 2007)(2, 3). However, few studies have been reported to investigate DPI translation on the basis of error analysis (EA). Based on Carl James’ error categorization, our study made an investigation into the distribution and potential causes of the common lexical errors in C-E translation of DPIs through identification, classification, description and diagnosis. On the basis of Eugene Nida’s translation theory, some strategies for C-E translation of vocabulary in DPIs were proposed to improve translation quality.

## STRUCTURE AND LEXICAL FEATURES OF DPIS

Most of the DPIs are composed of three parts: title or headline, body copy and ending. *Title* or *headline* is often in bold and big typeface in the middle of the first line. *Body copy* is the most important part of a DPI and can be divided into different sections, each with a subtitle. *Ending* generally covers such information as ratification number, manufacturer, addresses and contact information. According to the regulations from Food and Drug Administration (FDA), the body copy of a DPI should include the following sections in sequence:

1) *Drug name:* including generic name, trade name and chemical name;
2) *Description/Characteristics:* a description of the properties of the medicine, including the color, shape and form in which the drug is prepared;
3) *Composition/Components:* the qualitative and quantitative composition of the active substance and the full qualitative composition of the excipients;
3) *Composition/Components:* the qualitative and quantitative composition of the active substance and the full qualitative composition of the excipients
4) *Indications:* symptoms or particular circumstances which indicate the advisability or necessity of a specific medical treatment or procedure;
5) *Dosage and administration:* information about the usage and dosage of the drug in various situations;
6) *Adverse reactions:* side effects which are normally listed in decreasing order of seriousness;
7) *Contraindications:* those who are forbidden from using the drug, such as pregnant and nursing mothers or special patient populations;
8) *Precautions/Warnings:* information about the influence of the drug on the patients’ behavior, possible symptoms, intolerance of specific materials, etc;
9) *Drug interactions:* effects of food, alcohol and other drugs on the drug;
10) *Package/Presentation:* the size and form of the drug package;
11) *Storage:* the storage conditions, including the environment and temperature requirements;
12) *Validity/Expiry date:* the maximum in-use life of the drug.

In addition to the twelve sections, *body copy* of some DPIs consists of such sections as *pre-clinical tests on animals, clinical experience*, overdosage, etc.

A DPI is characterized by formality, objectivity, preciseness, conciseness and logicality. As a type of product description, vocabulary in a DPI shares the common features of medical texts but it also has some specific features. Abundant of technical and semi-technical terms are used in a DPI and the general words in a DPI should be easily understood by laymen. Besides, auxiliary verbs and modal verbs, such *as may, can, should, must*, are frequently used in some sections of DPIs like adverse reactions, contraindications, precautions, etc.

### C-E TRANSLATION CRITERION OF VOCABULARY FOR DPIS

The primary function of DPIs is *informative function*. A drug package insert conveys a bulk of relevant information on the drug and its administration.

According to Nida, translating was to reproduce “the closest natural equivalent” of the source language message in the receptor language, “first in terms of meaning and secondly in terms of style” (Nida and Taber, 2004)(4). Nida analyzed equivalence in terms of communicative functions of language and claimed that the informative function in language could only be served by a translation which was thoroughly understandable. Therefore, Chinese-English DPI translation aims at achieving the closest natural equivalence to the original in meaning, structure and style. The rendering of DPIs should not only convey accurate information but also present the message in a clear, smooth, concise and logical manner for communication. Based on the stylistic features of DPIs and the principle of “functional equivalence”, the criterion for C-E translation of DPIs should be accuracy in meaning, smoothness in expression and faithfulness in style.

## METHODS

### Corpus Establishment

**Collection of drug package inserts:** 60 C-E DPIs were collected for the establishment of the corpus in accordance with the criteria proposed by Nwogu 1997(5): representativity, reputation and accessibility. In terms of representativity, the collected samples included DPIs for both western medicines and Chinese patent drugs, either for external use or for oral administration. In the aspect of reputation, majority of the DPIs were for the drugs manufactured by some famous pharmaceutical factories in China, such as *Yunnan Baiyao Group Co., Ltd, Beijing Zizhu Pharmaceutical Co., Ltd, Yantai Rongchang Pharmaceutical Co., Ltd*, and *Beijing Tongrentang Limited Company,* etc. With regard to accessibility, all the DPIs were collected and accessible from many drugstores in China.

**Sampling of drug package inserts:** a three-round selection was conducted by stratified random sampling. 1) The selected DPIs with C-E translation should be for the drugs manufactured in China and used for treatment. Therefore, 24 DPIs for imported drugs or for health care products were excluded. 2) The length of the English version of each selected DPI should be no less than 150 words and the translation of a selected DPI should include at least six sections: drug name, description, composition, indications, dosage and administration, adverse reactions/contraindications/precautions, etc. Consequently, 2 DPIs were eliminated from the corpus. 3) 25 DPIs were randomly selected from the remaining 34 DPIs and thus a translation corpus was established.

### Data Collection and Processing

The errors in each sample DPI in the translation corpus were identified on the basis of British and American standard written English and translation criterion for DPIs. All the detected errors were categorized according to Carl James’ error taxonomy. Some experts in medicine or medical translation were also consulted for reference during the process of error identification and classification. The frequency of errors in different categories was counted manually, computed with Microsoft Office Excel 2003 and analyzed with SPSS16.0 software. Kruskal-Wallis *H* test and Wilcoxon rank sum test were conducted to compare the frequency of error occurrence in various categories and the alpha value was set up at 0.05.

## RESULTS AND DISCUSSION

The errors identified in this study were divided into three levels based on Carl James’ classification(1): substance errors, text errors and discourse errors, each with several subtypes. A total of 576 errors were detected in the study, including 54 substance errors, 446 text errors and 76 discourse errors as shown in Table 1.

**Table 1:** Frequency and percentage of errors at three levels (n=25)

Levels	Statistical Description			
	Frequency	Percentage	X ± S	SE

Substance	54	9.38%	2.16 ± 2.13^a^ ^b^	0.43
Text	446%	77.43%%	17.84 ± 10.19	2.04
Discourse	76	13.19%%	3.04 ± 2.68^a^	0.54
Total Number of Errors	576	100%	7.68 ± 9.48	1.09

a *P*<0.01 *vs.* text errors;

b *P*>0.05 *vs.* discourse errors.

At text level, grammar errors (270, 60.54%) outnumbered lexical ones (176, 39.46%) by 21.08 percent as shown in Table 2, but no significant difference was found between the two types of errors (*P*>0.05).

**Table 2:** Frequency and percentage of errors at text level (n=25)

Error Types	Statistical Description			
	Frequency	Percentage	X ± S	SE

Lexical Errors	176	39.46%	7.04 ± 4.11^a^	0.82
Grammar Errors	270	60.54%	10.80 ± 7.60	1.52
Total Number of Text Errors	446	100%	8.92 ± 6.34	0.89

a *P*>0.05 *vs.* grammar errors.

Although more grammar errors than lexical errors were identified in our study, the errors in morphology or syntax, such as errors in tenses, concord, word order, etc. do not impede the understandability of the translated DPIs. However, a minor lexical error will have a strong and decisive effect on the readability and understandability of the translation of DPIs because vocabulary, from which the message often has to be inferred, carries a particularly heavy functional load (Carl James, 2003)(1). Lexical errors in translation may lead to the patients’ misuse of the drug products and even cause their death. Therefore, this paper places its focus on the analysis of lexical errors in the translation of DPIs and the strategies of improving them.

### Distribution of Lexical Errors

**Figure 1:**
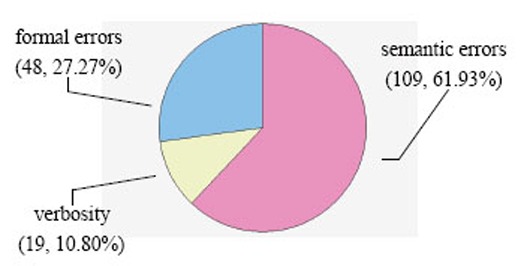
Frequency and percentage of lexical errors (n=25).

In lexical errors (see Figure 1), semantic errors (109, 61.93%) significantly exceeded formal errors (48, 27.27%) and verbosity (19, 10.80%) in frequency (*P*<0.01). The high frequency of semantic errors indicated that difficulties in semantics were the chief obstacle for many DPI translators in China to effective C-E translation of DPIs and the greatest challenge facing some intermediate-advanced English learners in China.

### Case Analysis of Lexical Errors

Some examples were cited from the corpus to illustrate the common lexical errors in C-E translation of DPIs. In the examples, the asterisk * represents erroneous translation, the tick √ indicates the corrected translation enclosed between parentheses (…), square brackets […] enclose a supplemented element, and angle brackets <…> enclose a redundant element.

**A. Formal Errors.** Formal errors consisted of formal misselection and misformations in the study. *Formal misselection* included errors of malapropism and confusions of part of speech. For example:

1) 
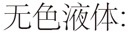
 a colorless *liquor (√liquid)

2) 
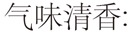
 *fragrance (√fragrant) smell

The error of malapropism in Example 1) was a misselection between a pair of synforms which share the same initial part and syllables. The feature of this type of errors is that the substitute is a word in the target language (TL) and resembles the target word in form but not necessarily in meaning. Confusion of part of speech in Example 2) was caused by the improper choice between a noun and an adjective deriving from the same root.

*Misformations* are errors that produce non-existent “words” in English, which can either originate in the source language (interlingual misformation errors) or be created from the target language (intralingual errors of form), including calque, borrowing and coinage. In Example 3), the coined “*post-diet” is not a standard English word.

3) 
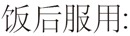
 be taken *post-diet (√after meals)

**B. Semantic Errors.** Lexicologists describe vocabulary in terms of a system of lexico-semantic clusterings known as semantic field or lexical field, which reflects the relations between related words and expressions, mainly consisting of paradigmatic relations and syntagmatic relations. The former refers to the relation between words or expressions that are replaceable with each other at a particular place in a structure, and the latter refers to the restrictions on how words can be used together. Errors in paradigmatic and syntagmatic relations are called *confusion of sense relations* and *collocational errors*, respectively.

Confusion of sense relations identified in the study included the improper use of superonym for hyponym, hyponym for superonym, whole for part, part for whole, antonym, misselection between two co-hyponyms, and misuse of near-synonyms, which are shown in the following examples:

4) 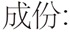 *ingredients (√composition)

5) 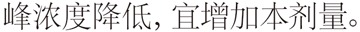 Dosage should be *reduced (√increased) when Cmax decreases.

6) 
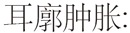
 *ear (√auricle) edema

7) 
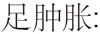
 *toe (√foot) swelling

8) 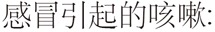 cough caused by *influenza (√cold)

9) 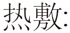 heat treatment (√hot compress)

Semantic error in Example 4) was caused by using the wrong word from a set of near-synonyms. Such synonyms are semantically similar or identical in Chinese but quite different in English. Although “composition” and “ingredient” can be translated into Chinese as “) 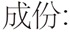
”, according to *Oxford English-Chinese Dictionary*, “ingredient” is any of the foods that are combined to make a particular dish, while “composition” means the parts of which something is made, such as “the composition of the soil”. In Example 5), an antonym “reduce” was used instead of “increase”. Examples 6) and 7) are errors of whole for part and part for whole respectively. Lexical error in Example 8) is a case of misusing two co-hyponyms “influenza” and “cold”, which are the hyponyms of the word “disease”. Error in Example 9) is due to the use of a general term instead of a specific one (superonym for hyponym), the result of which is the under-specification of the meaning. “Heat treatment” is a series of treatment including “hot compress”, “diathermy”, etc.

Collocational errors in the study occurred in two degrees: errors in semantically determined word selection and errors in combinations with statistically weighted preferences, which are shown in Examples 10) and 11), respectively.

10) 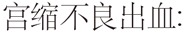 bleeding from *faulty (√abnormal) uterine constriction

11) 
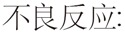
 *ill (√adverse) reactions

**C. Verbosity.** In the study verbosity referred to the use of more words in the target language than necessary in translation. For example:

12) 
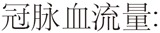
 *volume of coronary blood flow (√coronary blood flow or √CBF)

### Diagnostic Error Analysis

The causes for making lexical errors in C-E translation of DPIs are diagnosed in our corpus, ranging from the misunderstanding of the source text to the lack of proficiency in English language. There were three types of diagnosis-based errors: interlingual errors, intralingual errors and communication strategy-based errors.

### Interlingual Errors

Interlingual errors result from negative transfer from language, culture and thinking pattern of the mother tongue. When the elements of the translators’ mother tongue are similar to those of the target language, the translators benefit from positive first language (L1) transfer. On the contrary, when the elements are different, the translators are hindered by negative transfer or mother tongue interference, which results in interlingual errors.

In this study, interlingual errors in lexis involved errors in word meaning, collocation, part of speech and formation of phrases. When a word in Chinese has two or more different counterparts in English, some translators often fail to notice the distinction of word meaning between or among those counterparts in English. In Example 13), the Chinese word 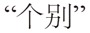 has two different meanings. When translated into English, the misselection between the two equivalents “individual” and “very few” led to an interlingual error of word meaning.

13) 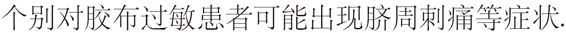 Twinge in the skin around the umbilicus may occur in *individual (√very few) patients allergic to the nonwoven tape.

Negative transfer in lexis also leads to errors of part of speech as shown in Example 14). In Chinese part of speech of a word can be the same no matter it is used as an adjective, an adverb, a verb or a noun. However, part of speech in English is reflected through inflectional changes of the word.

14) 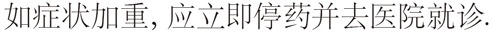 If the symptom is aggravated, *withdrawal (√withdraw) the drug or consult a doctor.

Since English and Chinese belong to quite different writing systems, interlingual errors caused by negative transfer occur not only in individual words but also in phrases. The remarkable feature of such errors made by Chinese translators is “word-for-word” translation, which reveals that some Chinese learners tend to attach too much attention to the literal meaning of individual words and ignore the exact meaning of the phrase as a whole. For instance:

15) 
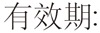
 *effective period (√shelf life/√validity).

16) 
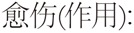
 *healing wounds effects (√wound healing effects).

17) 
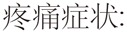
 *painful symptoms (√symptoms of pain).

### Intralingual Errors

Intralingual errors are due to the misapplication of the learning strategies for code-breaking, such as overgeneralization, ignorance of cooccurrence restrictions, incomplete rule application and redundancy. Among them, exploiting redundancy is the main cause of lexical errors in this study. Redundancy occurs when unnecessary information and double signaling are provided. In the study such an error reflected the insecurity on the translators’ part: overcompensation for the sense of linguistic inadequacy they harbored. The following sentence 18) is a typical example:

18) 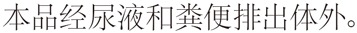 It is excreted *<out of the body> in the urine and stools.

The word “excrete” means “to get rid of waste material from one’s body through bowels, skin etc” so the phrase “out of the body” is unnecessary in translation.

### Communication Strategy-based Errors

This kind of errors included approximation, circumlocution and avoidance in the study. *Approximation* occurred when the translators replaced the required form with another near-equivalent item in TL they had learnt, such as a near synonym, a superordinate term, an antonym, a coined word, calque, etc. For example, the translator used a near synonym “breast” to translate 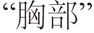 instead of “chest”.

*Errors of circumlocution* occurred when the translators referred to the entity with one or more criterial attributes of the referent. For example, 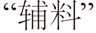 was translated into “*pharmaceutical additive” rather than “adjuvant”.

*Avoidance* was caused by the translators’ ignorance of the TL item, which resulted in lack of information load in the study. For example, 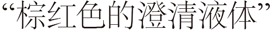 was translated into “reddish brown liquid”, in which 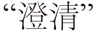 (clear *or* transparent) was omitted by the translator.

## STRATEGIES FOR C-E TRANSLATION OF VOCABULARY IN DPIS

The accurate translation of vocabulary matters a great deal to the readability and reliability of the translated DPIs. Therefore, it is very essential for translators to employ some strategies in translating vocabulary in DPIs to reduce lexical errors. Vocabulary used in DPIs is broadly classified into three types: technical terms, semi-technical terms and general words.

### Technical and Semi-technical Terms

**Figure 2:**
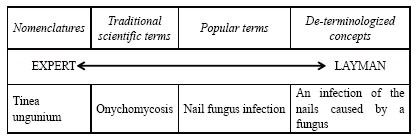
Ways of expressing specialized concepts.

Technical and semi-technical terms in DPIs are units of specialized knowledge for facilitating the organization and transmission of medical knowledge in specialized contexts. A lot of words in DPIs are technical and semi-technical terms closely connected with medicine, biology and chemistry. The translators’ major difficulty in translating the terms from Chinese into English is that there is only one concept in Chinese but a variety of synonyms in English. The multiple terms in English vary from completely internationalized nomenclatures to traditional scientific terms, from popular terms to de-terminologized concepts (Montalt & Davies, 2007). Figure 2 shows the translation of 
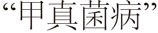
 into different English terms or words.

As shown in Figure 2, nomenclatures and traditional scientific terms are more discipline-bound and neutral and they are mainly used for communication between experts. Popular terms and de-terminologized concepts are more text-bound and culturally marked and they are chiefly used for expert-to-layman or layman-to-layman communication (Montalt & Davies, 2007)(3). Since DPIs are written by professionals for semi-professional or non-professional readers and mainly for expert-to-nonexpert communication, nomenclatures and de-terminologized concepts are seldom used. As a result, the principle of translating technical and semi-technical terms in DPIs is that traditional scientific terms and popular terms in Chinese should be rendered into their equivalent terms in English.

### General Words

General words make up a larger proportion of vocabulary in DPIs compared with technical and semi-technical terms. Most lexical errors identified in the study were attributable to the poor translation of general words. In C-E translation of general words in DPIs, much attention should be paid to their degree of formality and the relations between words.

**A. Degree of Formality.** Vocabulary used on different occasions differs in the degree of formality. Formal English words should be used instead of informal or slang words in translating general words in DPIs. A misselection from several general English words for a non-medical concept in Chinese deviates from the formal style of DPIs and is even offensive to the users. For instance, in the context of DPI translation, 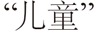 should be translated into “*children*” rather than “*kids*”, “*nippers*” or “*tykes*”.

**B. Paradigmatic and Syntagmatic Relations.** The paradigmatic relations between words play a vital role in C-E translation of general words in DPIs because the meaning of a word depends largely on its place in the semantic field. For example, 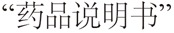 should be translated into “package insert” rather than “manual” for the reason that the former refers to a piece of paper inserted in the package along with the product used for short instructions while the latter is a book giving information about a product.

The syntagmatic relations or collocational relations between words are also important for translators to choose an appropriate word in C-E translation. Although a general word can be collocated with different words in Chinese, it should be translated into its appropriate equivalents based on the collocational restrictions in English. For example:

1) 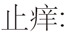 to *relieve* itching

2) 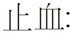 to *stop* bleeding

3) 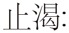 to *quench* thirst

Therefore, when the translators are uncertain about the choice of a general word in C-E translation of DPIs, they should make a thorough analysis of the differences between words in their usage by consulting some authoritative modern English-English dictionaries and then choose the exact word according to the context.

## CONCLUSIONS

Through the in-depth analysis of distribution and causes of the common lexical errors in C-E translation of DPIs based on Carl James’ EA methodology, we can draw the following conclusions.

Firstly, large quantities of lexical errors identified in the C-E translation corpus of DPIs are interlingual and intralingual errors so it is essential for translators to develop their ability to use language, especially their competence of using vocabulary. Intralingual errors of lexis are attributed to the translators’ faulty or partial knowledge of the lexical rules in English. Consequently, the translators should have a good command of English, particularly the semantic relations between words and the collocational restrictions of vocabulary, which will have a direct bearing on the quality of C-E translation. Interlingual errors of lexis are caused by interference of the translators’ native language so the translators should bridge the gap between Chinese and English by fully understanding their differences in languages, cultures and ways of thinking to avoid negative transfer from Chinese in C-E translation of vocabulary in DPIs.

Secondly, a lot of lexical errors identified in the study are closely associated with the stylistic features of a DPI, such as the use of informal words, redundant expression, and inappropriate use of modal verbs. Accordingly, the translators of a DPI should be not only equipped with the competence in both Chinese and English language but also acquainted with the specific structure and features of DPIs. In addition, translators should have basic medical knowledge, essential knowledge of translation theories and techniques, corresponding translation strategies for vocabulary and general cultural awareness.

Through the investigation of lexical errors in C-E translation of drug package inserts and the analysis of the underlying causes, some feasible translation strategies for correcting lexical errors have been proposed, which will be helpful for medical workers and translators to heighten their vigilance against the common lexical errors and develop their translating ability in C-E translation of DPIs and other types of medical texts.
